# Effect of Exogenous Hydrogen Sulfide and Polysulfide Donors on Insulin Sensitivity of the Adipose Tissue

**DOI:** 10.3390/biom12050646

**Published:** 2022-04-28

**Authors:** Jolanta Kowalczyk-Bołtuć, Krzysztof Wiórkowski, Jerzy Bełtowski

**Affiliations:** 1 Endocrinology and Metabolism Clinic, Internal Medicine Clinic with Hypertension Department, Medical Institute of Rural Health, 20-090 Lublin, Poland; kowalczyk-boltuc.jolanta@imw.lublin.pl; 2 Care Unit, Luxmed Lublin Medical Center—Primary, 20-080 Lublin, Poland; kwiorkowski@gmail.com; 3 Department of Pathophysiology, Medical University of Lublin, 20-090 Lublin, Poland

**Keywords:** adipose tissue, hydrogen sulfide, polysulfides, insulin sensitivity, obesity

## Abstract

Hydrogen sulfide (H_2_S) and inorganic polysulfides are important signaling molecules; however, little is known about their role in adipose tissue. We examined the effect of H_2_S and polysulfides on insulin sensitivity of the adipose tissue in rats. Plasma glucose, insulin, non-esterified fatty acids, and glycerol were measured after administration of H_2_S and the polysulfide donors, Na_2_S and Na_2_S_4_, respectively. In addition, the effect of Na_2_S and Na_2_S_4_ on insulin-induced glucose uptake and inhibition of lipolysis was studied in adipose tissue explants ex vivo. Na_2_S and Na_2_S_4_ administered in vivo at a single dose of 100 μmol/kg had no effect on plasma glucose and insulin concentrations. In addition, Na_2_S and Na_2_S_4_ did not modify the effect of insulin on plasma glucose, fatty acids, and glycerol concentrations. Na_2_S and Na_2_S_4_had no effect on the antilipolytic effect of insulin in adipose tissue explants ex vivo. The effect of insulin on 2-deoxyglucose uptake by adipose tissue was impaired in obese rats which was accompanied by lower insulin-induced tyrosine phosphorylation of IRS-1 and Akt. Na_2_S_4_, but not Na_2_S, improved insulin signaling and increased insulin-stimulated 2-deoxyglucose uptake by adipose tissue of obese rats. The results suggest that polysulfides may normalize insulin sensitivity, at least in the adipose tissue, in obesity/metabolic syndrome.

## 1. Introduction

In 1996, Abe and Kimura first demonstrated that hydrogen sulfide (H_2_S) is endogenously produced in mammalian tissues and serves as the new gasotransmitter together with its older counterparts, nitric oxide (NO) and carbon monoxide (CO) [1]. Since that time, H_2_S has been increasingly recognized as an important mediator involved in the regulation of cardiovascular, nervous, gastrointestinal, renal, immune, endocrine, and other systems. In addition, deficiency or excess of H_2_S has been implicated in the pathogenesis of different diseases such as arterial hypertension, atherosclerosis, ischemic heart disease, heart failure, peptic ulcer disease, cancer, neurodegenerative disease, inflammation, etc. [2,3,4,5,6,7,8]. Subsequently, Kimura et al. demonstrated that not only H_2_S but also inorganic polysulfides (H_2_S_n,_ n = 2–8) are produced in tissues and serve as the important mediators with some effects similar but other distinct that H_2_S [9,10,11]. For example, polysulfides can directly react with protein thiols (−SH) to convert them to hydropersulfide (−SSH) groups; the process is referred to as protein persulfidation. In contrast, the reaction between H_2_S and protein −SH groups requires their prior modification to disulfides, sulfenic acid, or S-nitrosothiols [12,13].

Excess adipose tissue (obesity) is one of the most prevalent morbidities nowadays. Obesity, mainly associated with consuming energetically dense food and a sedentary lifestyle, is associated with many complications such as dyslipidemia, diabetes, cardiovascular disease, osteoarthritis, neurodegenerative disease, and certain types of cancer [14,15,16]. Insulin resistance is a hallmark of obesity-associated pathologies and is suggested to be involved in the pathogenesis of many obesity-associated complications [17]. On the other hand, adipose tissue is one of the main insulin target tissues in addition to skeletal muscles and the liver [18].

In comparison to other systems, the role of H_2_S in adipose tissue has not been extensively studied so far. H_2_S has been demonstrated to be involved in the regulation of adipogenesis, lipolysis, adipokine production, etc., but the results of studies on these topics are often controversial [19,20]. The role of H_2_S in the regulation of insulin signaling is also ambiguous; both stimulatory [21] and inhibitory [22] effects of H_2_S donors on insulin-induced glucose uptake in adipose tissue have been described. Recently, we have demonstrated that H_2_S stimulates adipose tissue lipolysis in the adipose tissue in a cAMP-dependent manner and that H_2_S production in the adipose tissue is increased in rats with short-term obesity in which lipolysis is also stimulated [23]. Adipose tissue lipolysis is strictly regulated by many hormones and mediators [24]. Insulin inhibits lipolysis by stimulating phosphodiesterase and decreasing intracellular cAMP concentration. The enhanced adipose tissue lipolysis characteristic of obesity could result from insulin resistance but also may contribute to insulin resistance due to the detrimental effect of excess fatty acids on insulin signaling. Although the H_2_S donor, Na_2_S, increased cAMP in the adipose tissue [23], it is unclear if this effect resulted from inducing insulin resistance. Therefore, in the present study, we examined the effect of H_2_S and polysulfides on insulin signaling in adipose tissue as well as on insulin-induced inhibition of lipolysis and insulin-induced stimulation of glucose uptake.

## 2. Materials and Methods

### 2.1. Reagents and Animals

Until otherwise stated, all reagents were obtained from Sigma-Aldrich (Steinheim, Germany). Na_2_S and Na_2_S_4_ solutions were prepared immediately before use. Sulfide salts were dissolved in phosphate-buffered saline (pH 7.4) containing 100 μM of a metal chelator, ditehylenetriaminepentaacetic acid (DTPA), previously deoxygenated by bubbling with N_2_ for 15 min to prevent sulfide oxidation.

All experiments were performed on 56 young-adult (2- to 2.5-month-old) male Wistar rats weighing 200–230 g. The study was approved by the Local Ethical Committee in Lublin (approval number 19/2019). Animals were kept at a temperature of 20 ± 2 °C under a 12 h light–dark cycle and had free access to chow and tap water. One group of rats (control lean) was fed standard rodent chow (68% carbohydrates, 20% protein, and 12% fat, Agropol, Motycz, Poland), whereas the second group received a high-calorie diet containing standard chow and a mixture of milk powder, sucrose, glucose, and soybean powder (1:1:1:1) [25]. The composition of this diet (percentage of calories derived from carbohydrates, proteins, and fat) is similar to that of normal chow, but the diet is highly palatable and increases food intake. The animals were fed both diets for 1 month.

For the in vivo experiments (performed only in lean rats), animals were anesthetized with ethylurethane (1.25 g/kg ip.) A thin polyethylene cannula (World Precision Instruments, Sarasota, FL, USA) was inserted into the carotid vein for continuous infusion of physiological saline (2 mL/h) to avoid hypovolemia. The second cannula was inserted into the carotid artery for blood sampling. Body temperature was monitored by a rectal thermometer (World Precision Instruments, Sarasota, FL) and was maintained at 36.5–37.5 °C using a heating table.

### 2.2. Effect of Na_2_S and Na_2_S_4_ on Plasma Insulin and Glucose Concentrations

In the first set of experiments, we examined the effect of the H_2_S donor, Na_2_S, and the polysulfide donor, Na_2_S_4_, on plasma insulin and glucose concentrations. After a 30 min stabilization period, the baseline blood sample was collected. Next, 0.5 mL of 0.9% NaCl (control), Na_2_S or Na_2_S_4_ was administered intravenously at 100 μmol/kg in 0.5 mL 0.9% NaCl). Blood samples (0.5 mL) were collected after 15, 30, 45, and 60 min into tubes containing EDTA and were centrifuged at 2000× *g* for 5 min. Plasma was frozen and stored at −80 °C until the assay.

### 2.3. Effect of Insulin on Plasma Glucose, Non-Esterified Fatty Acids (NEFA), and Glycerol and Its Modification by Na_2_S and Na_2_S_4_

The experiments were performed in following groups of rats receiving: (1) 0.5 mL 0.9% NaCl intravenously and after 15 min 0.5 mL 0.9% NaCl intraperitoneally, (2) 0.5 mL 0.9% NaCl intravenously and after 15 min insulin (0.5 U/kg) intraperitoneally, (3) Na_2_S (100 μmol/kg) intravenously and after 15 min insulin (0.5 U/kg) intraperitoneally, (4) Na_2_S_4_ (100 μmol/kg) intravenously and after 15 min insulin (0.5 U/kg) intraperitoneally, (5) Na_2_S (100 μmol/kg) intravenously and after 15 min 0.5 mL 0.9% NaCl intraperitoneally, and (6) Na_2_S_4_ (100 μmol/kg) intravenously and after 15 min 0.5 mL 0.9% NaCl intraperitoneally. Blood samples for the measurement of glucose, NEFA, and glycerol were obtained 15, 30, 45, and 60 min after the second injection. In addition, 30 min after the second injection, slices of mesenteric adipose tissue were excised for the measurement of cAMP and proteins involved in the insulin signaling.

### 2.4. Effect of Insulin, Na_2_S, and Na_2_S_4_ on Insulin-Induced Glucose Uptake and Lipolysis Ex Vivo

Pieces of mesenteric adipose tissue (100 mg) were collected from lean and obese rats under general anesthesia in ice-cold Tyrode solution (140 mM NaCl, 5 mM KCl, 2 mM CaCl_2_, 1.1 mM MgCl_2_, 10 mM HEPES, and 5.5 mM glucose, pH 7.4) saturated with 21% O_2_/5% CO_2_; 2–4 pieces per 2 mL Eppendorf tube) and incubated at 37 °C for 15 min for stabilization. Then, adipose tissue slices were moved to other tubes and were incubated for 15 min in Tyrode solution containing 4.5 mM glucose and 1 mM 2-deoxyglucose (2-DG) with or without insulin (10 mU/mL), Na_2_S (100 μM) or Na_2_S_4_ (100 μM). 2-DG uptake was measured by the bioluminescent method [26]. 2-DG is taken up by the cells and then phosphorylated to 2-deoxyglucose-6-phosphate (2-DG6P) by hexokinase which is not further metabolized. The method is based on the detection of NADPH originating during glucose 6-phosphate dehydrogenase-catalyzed oxidation of 2-DG6P in the presence of NADP^+^. NADPH reduces proluciferin in the presence of reductase and luminescence of luciferin in the presence of luciferase is measured.

After incubation, adipose tissue was homogenized in 2% dodecyl trimethylammonium bromide (DTAB) in 0.4 M HCl (1.0 mL/100 mg tissue) to stop glucose uptake and destroy NADPH present in the cells, and the homogenate was centrifuged at 10,000× *g* for 10 min at 4 °C. Supernatant (25 μL) was added to microplate wells together with 25 μL Tris buffer (pH 10.0). Then 100 μL of Glo^TM^ reagent (Promega Corporation, Madison, WI) containing proluciferin, reductase, and luciferase with additionally added 20 μM NADP^+^ and 2.5 U/mL glucose-6-phosphate dehydrogenase from *S. cerevisiae*. Luminescence was measured after 30 min using a microplate reader (PHERAstar FS, BMG Labtech, Ottenberg, Germany). The standard curve was prepared using various concentrations (0–50 μM) of 2-DG6P. All steps since mixing with DTAB in the HCl solution described above were performed with the standards. The results are expressed in pmol 2-DG/mg adipose tissue.

The effect of insulin, Na_2_S, and Na_2_S_4_ on adipose tissue lipolysis was examined by measuring glycerol concentration in the medium after 15 min of incubation.

### 2.5. Phosphorylation of Insulin Receptor β Subunit (IRβ)

IRβ phosphorylation was measured by the ELISA method using the PathScan Phospho-Insulin Receptor β (Tyr1150/1151) kit (Cell Signaling Technology, Danvers, MA, USA). Slices of mesenteric adipose tissue were homogenized in lysis buffer containing 20 mM Tris (pH 7.4), 150 mM NaCl, 1 mM EDTA, 1 mM EGTA, 1% Triton X-100, 2.5 mM sodium pyrophosphate, 1 mM glycerol 2-phosphate, 1 mM Na_3_VO_4_, 1 μg/mL leupeptin, 1 mM phenylmethylsulfonyl fluoride, and centrifuged at 10,000× *g* for 10 min. The supernatant was diluted to 0.025 protein/mL and pipetted into microplate wells coated with antibodies specific for IRβ irrespective of its phosphorylation. After incubation and washing, 50 μL of rabbit immunoglobulins specific for IRβ phosphorylated at Tyr^1150^ were added followed by 50 μL of anti-rabbit IgG conjugated with horseradish peroxidase and 50 μL of substrate (trimethylbenzidine+H_2_O_2_) solution. After 15 min, absorbance was read at 450 nm. The results were expressed as the ratio of optical density in the test sample and the mean optical density in the control group. The intra- and inter-assay CVs were <6% and <8%, respectively.

### 2.6. Phosphorylation of Insulin Receptor Substrate-1 (IRS-1)

Total IRS-1 and IRS-1 phosphorylated at tyrosine residues were measured using PathScan Total IRS-1 and Phospho-IRS-1 (panTyr) kits, respectively (Cell Signaling Technology, Danvers, MA, USA). Fifty microliters of adipose tissue homogenate supernatant (see Section 2.5) was added to the microplate wells coated with anti-IRS-1 antibodies. After incubation and washing, 50 μL of mice anti-IRS-1 or anti-phosphotyrosine antibodies were added and then 50 μL of antibodies specific for mice IgG conjugated with horseradish peroxidase were pipetted to the wells. Then, 100 μL of TMB+H_2_O_2_ substrate was added and absorbance was measured at 450 nm after 15 min. Intra-/inter-assay CVs for total and phosphorylated IRS-1 were 5%/8% and 6%/9%, respectively. The results are expressed as the ratio between optical density for phosphorylated IRS-1 to optical density for total IRS-1 in a given sample.

### 2.7. Akt Phosphorylation

Protein kinase Akt is activated in response to insulin by phosphorylation of its Thre^308^ and Ser^473^ residues. We measured total Akt and Akt phosphorylated at Ser^473^ using Cell Signaling Technology PathScan Total Akt1 Sandwich ELISA Kit (Kit #7170) and PathScan Phospho-Akt1 (Ser473) Sandwich ELISA Kit (#7160), respectively. Adipose tissue slices were homogenized in 10 mM Tris (pH 7.4) containing 100 mM NaCl, 1 mM EDTA, 1 mM EGTA, 1 mM NaF, 20 mM Na_4_P_2_O_7_, 2 mM Na_3_VO_4_, 1% Triton X-100, 10% glycerol, 0.1% sodium dodecylsulfate, 1 mM pjhenylmethylsulfonyl fluoride, and a protease inhibitor cocktail (Sigma-Aldrich, cat. No. P2714, 250 μL/5 mL buffer). Homogenate was centrifuged at 14,000× *g* for 10 min at 4 °C and supernatant was diluted 50-fold. One hundred microliters of the diluted supernatant was pipetted to the wells coated with anti-Akt1 antibodies. The plate was incubated for 2 h at room temperature. After washing, 100 μL of anti-Akt1 antibodies conjugated with biotin were added and the plate was incubated for 1 h. After the next wash, 100 μL of streptavidin coupled with horseradish peroxidase was added and the plate was incubated for 30 min. Then, the plate was washed and 100 μL of TMB+H_2_O_2_ substrate solution was added to the wells. The plate was incubated for 30 min in the dark, the reaction was stopped by adding 100 μL of 0.5 M H_2_SO_4_, and absorbance was measured at 450 nm. Total Akt1 concentration was calculated from the standard curve (0–20 ng Akt/mL). The sensitivity of the method is 0.1 ng/mL whereas intra- and inter-assay CVs are 5.3% and 7.2%, respectively.

The same supernatant was used for the measurement of Akt1 phosphorylated at Ser^473^. In this kit, the plate is coated with the same anti-Akt1 antibodies specific for the enzyme regardless of its phosphorylation level whereas antibodies added after the first wash are specific only for Akt1 phosphorylated at Ser^473^ regardless of its phosphorylation at Thr^308^. These antibodies do not react with non-phosphorylated Akt1 or with Akt1 phosphorylated only at Thr^308^. Standard solutions (0–100 U/mL) contain Akt1 phosphorylated in vitro. One unit is defined as the amount of phosphor-Akt obtained following complete phosphorylation of 100 pg of the enzyme. The sensitivity of the method is 0.8 U/mL whereas the intra- and inter-assay CVs are 5.3% and 7.5%, respectively. The results are expressed in U/ng.

Although according to manufacturers’ data, the antibodies used in PathScan® Total Akt1 Sandwich ELISA Kit and PathScan® Phospho-Insulin Receptor β (Tyr1150/1151) Sandwich ELISA Kit are primarily designed to detect human and mouse proteins, we confirmed their usefulness in the rat by several approaches. First, we found no absorbance if homogenate was processed without protease inhibitors. Second, in contrast to adipose tissue homogenates, the respective proteins were not detected in the rat plasma. Third, Akt1 was detected in cytosolic fraction but not in membrane/microsomal fraction separated by 100,000× *g* centrifugation. Fourth, we observed a linear relationship between total protein concentration and Akt1 and phosphorylated insulin receptors in serially diluted samples. Fifth, we confirmed the reactivity of antibodies contained in the total Akt1 kit by generating the standard curve using recombinant rat protein. Finally, an increase in IRβ phosphorylation and no change in the amount of total Akt1 after insulin treatment confirm that the kit may be applied in rat experiments.

### 2.8. Measurement of cAMP in the Adipose Tissue

Adipose tissue slices were homogenized in 50 mM NaCl buffered with a phosphate buffer (pH 7.4) containing 10 μM of the phosphodiesterase inhibitor 3-isobutyl-1-methyl-xanthine (IBMX) to inhibit cAMP hydrolysis during sample processing (100 μL of buffer per 10 mg tissue). The homogenate was centrifuged at 14,000× *g* for 10 min at 4 °C and diluted 100-fold. Cyclic AMP was measured immunoenzymatically by Cayman Chemical kits (Ann Arbor, MI, USA, cat.# 581001). The sensitivity, intra-assay CV, and inter-assay CV were 0.1 pmol/mL, 5.1%, and 7.0%, respectively.

### 2.9. Insulin, Glucose, NEFA, and Glycerol Measurements

Plasma insulin was measured immunoenzymatically using the Mercodia kit (cat. #10-1250-01). The sensitivity, intra-assay, and inter-assay CV values for insulin measurement were 0.15 μg/L, 3.1%, and 4.4%, respectively. The anti-insulin antibodies contained in the kit exhibited 7% cross-reactivity with rat proinsulin and 0.001% cross-reactivity with rat C peptide. Plasma glucose was measured by the glucose oxidase method using a kit purchased from Alfa Diagnostics (Warsaw, Poland).

Plasma non-esterified fatty acids and glycerol in plasma and adipose tissue media were measured as previously described [23] using kits provided by Cayman Chemical (Ann Arbor, MI, cat. #700310 and 10010755, respectively).

### 2.10. Statistical Analysis

The results are expressed as the means ± SD from 6 animals/adipose tissue samples per group. Between-group comparisons were performed by Student’s t-test or ANOVA, followed by Tukey’s test for 2 groups and >2 groups, respectively. The results from the same group obtained at different time points were analyzed by ANOVA for related variables. A *p* < 0.05 was considered statistically significant.

## 3. Results

### 3.1. Effect of Na_2_S and Na_2_S_4_ on Plasma Insulin and Glucose Concentrations

**Figure 1:**
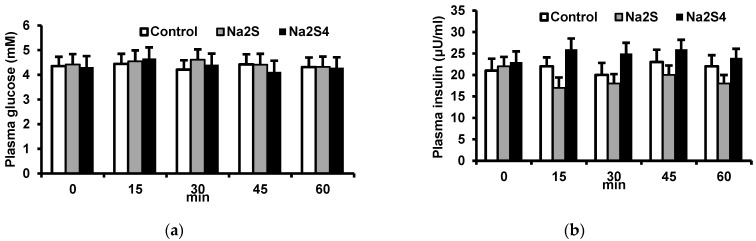
Effect of Na_2_S and Na_2_S_4_ on plasma glucose (**a**) and insulin (**b**) concentrations. Na_2_S or Na_2_S_4_ were injected intravenously and blood samples were collected before as well as 15, 30, 45, and 60 min after injection.

Neither Na_2_S nor Na_2_S_4_ had any effect on plasma glucose concentration (Figure 1).

### 3.2. Effect of Insulin on Plasma Glucose, NEFA, and Glycerol and Its Modification by Na_2_S and Na_2_S_4_

**Figure 2:**
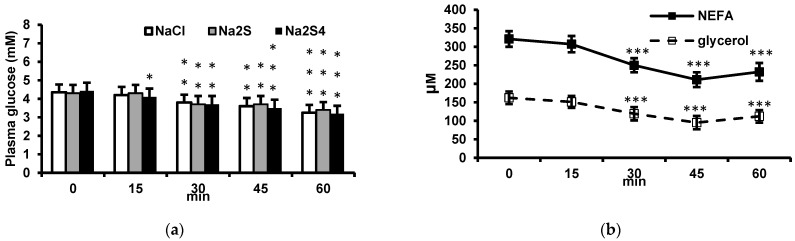
(**a**) Effect of insulin on plasma glucose concentration. Insulin was injected intraperitoneally at 0.5 U/kg and glucose was measured after 15, 30, 45 and 60 min. Rats received 0.5 ml 0.9% NaCl, 100 μmol Na_2_S or 100 μmol Na_2_S_4_ intravenously 15 min before insulin. (**b**) Effect of insulin on plasma NEFA and glycerol at different time points after injection. * *p* < 0.05, ** *p* < 0.01, *** *p* < 0.001 vs. baseline value in the respective group (repeated-measures ANOVA).

Insulin decreased plasma glucose concentration in a time-dependent manner at 15, 30, 45, and 60 min after injection. Na_2_S or Na_2_S_4_ administered before insulin had no effect on glucose levels in insulin-treated rats (Figure 2a).

Insulin decreased plasma NEFA and glycerol at 30, 45, and 60 min after injection. A maximal decrease in NEFA and glycerol was observed 45 min after insulin administration. NEFA and glycerol concentrations 45 min after insulin injection were lower by 34.3% and 48.8%, respectively, than in control rats (Figure 2b). These results confirm that insulin inhibits lipolysis in adipose tissue.

**Figure 3:**
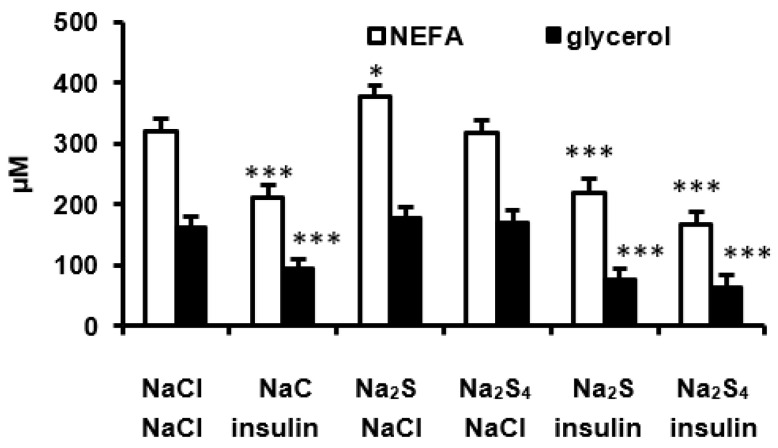
Effect of Na_2_S and Na_2_S_4_ (both at 100 μmol/kg) administered 15 min before insulin (0.5 U/kg) on plasma non-esterified fatty acids and glycerol 45 min after insulin administration. * *p* < 0.05, *** *p* < 0.001 vs. control group receiving two NaCl injection.

Then, we examined the effect of Na_2_S and Na_2_S_4_ administered 15 min before insulin on the antilipolytic effect of this hormone (Figure 3). NEFA and glycerol were measured 45 min after insulin injection when the maximal effect on lipolysis was observed. Na_2_S administered without insulin induced a small increase in NEFA but had no effect on glycerol concentration. In contrast, Na_2_S_4_ had no effect on NEFA or glycerol in rats not receiving insulin. NEFA and glycerol in rats injected with both Na_2_S or Na_2_S_4_ and insulin did not differ from after insulin alone. These results indicate that Na_2_S and Na_2_S_4_ do not modify the antilipolytic effect of insulin in vivo (Figure 3).

### 3.3. Effect of Insulin, Na_2_S, and Na_2_S_4_ on Insulin Signaling and cAMP in the Adipose Tissue In Vivo

**Figure 4:**
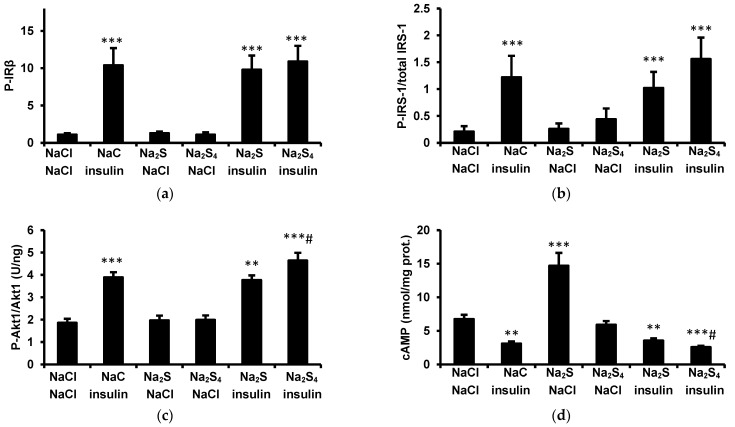
Effect of insulin, Na_2_S, and Na_2_S_4_ on phosphorylation of insulin receptor β-subunit (**a**), phosphorylation of IRS-1 (**b**), Akt1 phosphorylation at Ser^473^ (**c**), and cAMP in the adipose tissue (**d**) in vivo. NaCl (control), Na_2_S (100 μmol/kg), or Na_2_S_4_ (100 μmol/kg) were administered iv. 15 min before insulin (0.5 U/kg ip.). Samples of mesenteric adipose tissue were collected 30 min after insulin injection. ** *p* < 0.01, *** *p* < 0.001 vs. control group receiving only NaCl, # *p* < 0.05 vs. group receiving only insulin.

Insulin increased tyrosine phosphorylation of IRβ (Figure 4a), tyrosine phosphorylation of IRS-1 (Figure 4b), Akt1 phosphorylation at Ser^473^ (Figure 4c), and reduced cAMP concentration in the adipose tissue (Figure 4d). Na_2_S administered to rats not receiving insulin increased cAMP in the adipose tissue but had no effect on IRβ, IRS-1, and Akt1 phosphorylation. Na_2_S_4_ administered alone had no significant effect on the phosphorylation of insulin signaling proteins or cAMP. IRβ, IRS-1, and Akt1 phosphorylation, as well as the cAMP concentration in the adipose tissue, did not differ between rats treated with insulin and those receiving insulin and Na_2_S. Na_2_S_4_ administered before insulin had no effect on phosphorylation of IRβ and IRS-1, however, Akt1 phosphorylation was slightly higher and cAMP concentration was lower in rats receiving Na_2_S_4_ and insulin than in those receiving only insulin. These results suggest that Na_2_S_4_ augments insulin signaling in the adipose tissue.

### 3.4. Effect of Insulin, Na_2_S, and Na_2_S_4_ on Insulin-Induced Glucose Uptake in Adipose Tissue Ex Vivo

H_2_S and polysulfide donors administered intravenously can modulate adipose tissue metabolism indirectly, by affecting the level of hormones and mediators produced outside this tissue. In addition, H_2_S and H_2_S_n_ can be metabolized in vivo to other reactive sulfur species (RSS) such as sulfite with possible different activities [27]. To get more insight into the direct effects of H_2_S and H_2_S_n_ on adipose tissue metabolism, we performed additional experiments in which mesenteric adipose tissue slices were treated with insulin, Na_2_S, and/or Na_2_S_4_ ex vivo. For this purpose, adipose tissue was collected from rats fed either a standard diet or a high-calorie diet for 1 month.

Body weight, plasma NEFA, and glycerol concentrations were higher in obese than in lean rats, however, triglycerides, total cholesterol, glucose, and insulin concentrations did not differ between these groups (Table 1). These results indicate that the model used by us represented mild obesity without dyslipidemia, hyperglycemia, or insulin resistance, although higher NEFA and glycerol suggest enhanced adipose tissue lipolysis.

**Figure 5:**
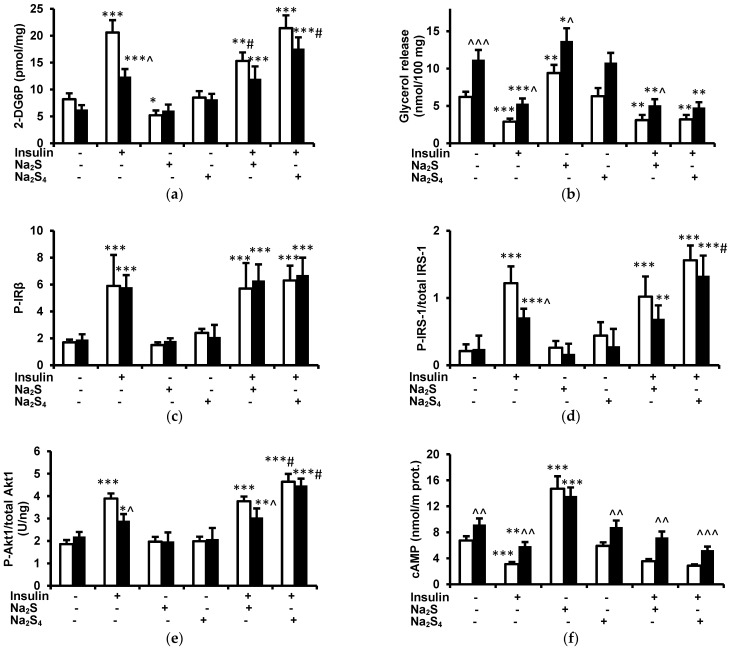
Effect of insulin, Na_2_S, and Na_2_S_4_ on glucose uptake (**a**), glycerol release (**b**), tyrosine phosphorylation of IRβ (**c**), tyrosine phosphorylation of IRS-1 (**d**), Akt1 phosphorylation (**e**), and cAMP concentration (**f**) in mesenteric adipose tissue isolated from lean (white) and obese (black) rats. * *p* < 0.05, ** *p* < 0.0, *** *p* < 0.001 vs. samples not treated with insulin, Na_2_S, and Na_2_S_4_ collected from the respective group of rats (lean or obese), ^ *p* < 0.05, ^^ *p* < 0.01, ^^^ *p* < 0.001 vs. adipose tissue samples treated in the same manner collected from lean rats, # *p* < 0.05 vs. samples from the respective group (lean or obese) treated with insulin alone.

Glucose uptake in the absence of insulin did not differ significantly between lean and obese rats. Insulin induced a significant increase in glucose uptake in both groups but the effect in the obese group was reduced in comparison to lean rats. Na_2_S without insulin decreased glucose uptake in the absence of insulin in lean rats but had no effect in obese rats. Na_2_S_4_ without insulin had no effect on glucose uptake in either the lean or obese group. Glucose uptake by adipose tissue isolated from lean rats in the presence of insulin and Na_2_S was lower than in the presence of insulin alone, however, because Na_2_S itself reduced baseline glucose uptake, relative insulin-induced increase in glucose uptake was similar in the absence and in the presence of Na_2_S (151.2 ± 20.2% and 194.2 ± 22.1%, respectively). Na_2_S had no effect on glucose uptake in the presence of insulin in obese rats. Na_2_S_4_ did not modify insulin-induced glucose uptake in lean rats, however, improved it in obese animals (Figure 5a).

### 3.5. Effect of Insulin, Na_2_S, and Na_2_S_4_ on Glycerol Release by Adipose Tissue Ex Vivo

Glycerol release in the absence of insulin was higher in obese than in lean rats. Insulin reduced glycerol release in lean and obese rats by 53.2% and 52.7%, respectively. These results suggest that the antilipolytic effect of insulin is preserved in obese rats, although glycerol release in the presence of insulin remained higher in obese rats. Na_2_S stimulated lipolysis in both lean and obese groups whereas Na_2_S_4_ had no effect. Glycerol release in the presence of insulin and Na_2_S or insulin and Na_2_S_4_ was similar to that in the presence of insulin alone in both lean and obese groups (Figure 5b).

### 3.6. Effect of Insulin, Na_2_S, and Na_2_S_4_ on Tyrosine Phosphorylation of Insulin Receptor β Subunit in Adipose Tissue Ex Vivo

In the absence of insulin, tyrosine phosphorylation of IRβ was similar in lean and obese rats. Insulin increased IRβ phosphorylation to a similar extent in both groups. Neither Na_2_S nor Na_2_S_4_ had any effect on IRβ phosphorylation in the absence of insulin and did not modify the stimulatory effect of this hormone (Figure 5c).

### 3.7. Effect of Insulin, Na_2_S, and Na_2_S_4_ on Tyrosine Phosphorylation of Insulin Receptor Substrate-1 in Adipose Tissue Ex Vivo

Tyrosine phosphorylation of IRS-1 in the absence of insulin was similar in lean and obese groups. Insulin increased IRS-1 phosphorylation in adipose tissue collected from lean and obese rats, however, IRS-1 phosphorylation in the presence of insulin was significantly lower in obese than in lean animals. Na_2_S had no effect on IRS-1 phosphorylation in the absence or in the presence of insulin. Na_2_S_4_ without insulin had no effect on IRS-1 phosphorylation. IRS-1 phosphorylation in adipose tissue of lean rats in the presence of insulin and Na_2_S_4_ tended to be higher than in the presence of insulin alone but the difference was not significant. In contrast, IRS-1 phosphorylation in adipose tissue of obese rats in the presence of insulin and Na_2_S_4_ was significantly higher than in the presence of insulin alone and became similar to that in lean animals. These data suggest that Na_2_S_4_ augments insulin-induced IRS-1 phosphorylation in obese rats (Figure 5d).

### 3.8. Effect of Insulin, Na_2_S, and Na_2_S_4_ on Akt1 Phosphorylation at Ser^473^ in Adipose Tissue Ex Vivo

Akt1 phosphorylation in adipose tissue in the absence of insulin was similar in lean and obese rats. Insulin increased Akt1 phosphorylation in both groups although the effect was impaired in obese animals. Na_2_S and Na_2_S_4_ had no effect on Akt1 phosphorylation in the absence of insulin in lean and obese groups. In addition, Akt1 phosphorylation in the presence of Na_2_S and insulin did not differ from Akt1 phosphorylation in the presence of insulin without Na_2_S in lean and obese rats. However, Akt1 phosphorylation in the presence of Na_2_S_4_ and insulin was higher than in the presence of insulin alone in lean and obese rats by 19.3% and 54.5%, respectively. Akt1 phosphorylation in the presence of insulin and Na_2_S_4_ did not differ between lean and obese groups (Figure 5e).

### 3.9. Effect of Insulin, Na_2_S, and Na_2_S_4_ on cAMP Concentration in the Adipose Tissue

Cyclic AMP concentration in the adipose tissue in the absence of insulin was higher in obese than in lean rats. Insulin decreased cAMP concentration in both groups although cAMP in the presence of insulin was still higher in obese than in lean animals. Na_2_S increased cAMP in the adipose tissue of both lean and obese rats, and cAMP after Na_2_S treatment became similar in lean and obese rats. Na_2_S_4_ had no effect on cAMP in either lean or obese groups. cAMP concentration in the presence of Na_2_S and insulin or Na_2_S_4_ and insulin did not differ from cAMP concentration after treatment with insulin without H_2_S/polysulfide donors in both lean and obese groups (Figure 5f).

## 4. Discussion

The main findings of this study are that: (1) a single dose of Na_2_S or Na_2_S_4_ has no effect on fasting glucose and insulin concentrations, (2) Na_2_S and Na_2_S_4_ have no effect on the insulin-induced decrease in plasma glucose, (3) Na_2_S and Na_2_S_4_ do not modify the antilipolytic effect of insulin either in vivo or ex vivo, (4) Na_2_S reduces glucose uptake by adipose tissue of lean but not obese rats both in the absence and in the presence of insulin ex vivo, (5) Na_2_S_4_ increases glucose uptake by the adipose tissue of obese rats in the presence but not in the absence of insulin, and (6) Na_2_S_4_ increases insulin signaling in the adipose tissue, especially in obese rats.

The role of H_2_S in the regulation of glucose metabolism, insulin secretion, and insulin sensitivity is controversial [28]. H_2_S has been demonstrated to inhibit glucose-induced insulin secretion in pancreatic β cells by activating ATP-sensitive K^+^ channels [29,30,31]. However, other authors observed a stimulatory effect of H_2_S on insulin synthesis [32]. In addition, both stimulatory [33] and inhibitory [34] effects of H_2_S on the secretion of glucagon-like peptide-1 (GLP-1), the important incretin stimulator of insulin release, have been observed. The present study suggests that both H_2_S and H_2_S_4_ have no major role in the regulation of insulin secretion, at least in the short run. However, our study was performed after the overnight fast and the regulation of insulin secretion differs in fasting and postprandial states. It is possible that reactive sulfur species are more important in the regulation of postprandial and/or glucose-stimulated than baseline insulin secretion. It should also be considered that locally produced H_2_S and/or H_2_S_4_ within islets could be more important in the regulation of insulin secretion than systemically administered donors.

The finding that Na_2_S and Na_2_S_4_ had no effect on either insulin or glucose levels suggests that these donors have also no major effect on insulin sensitivity, at least in the fasting state. The effect of insulin on fasting glucose is mainly mediated by inhibition of gluconeogenesis and hepatic glucose output. Several studies demonstrated that H_2_S donor, NaHS, stimulated, whereas knockout of H_2_S-synthesizing enzyme, cystathionine γ-lyase (CSE), inhibited gluconeogenesis in hepatocytes [35,36]. In addition, fasting plasma glucose was higher in high-fat diet-fed CSE knockout than in high-fat diet-fed wild-type mice [36]. NaHS administered at 39 μmol/kg increased fasting glucose and the expression of enzymes involved in gluconeogenesis in the liver of CSE knockout mice [37]. The difference between that study and our data may result from using different rodent species or various H_2_S donors (NaHS vs. Na_2_S). In addition, CSE deficiency might have an H_2_S-independent effect. The effect of reactive sulfur species in the regulation of gluconeogenesis in the liver remains to be studied in the future.

Cai et al. [21] have demonstrated that H_2_S-saturated buffer administered at a dose of 100 μmol/day improves glucose tolerance and augments the glucose-lowering effect of insulin in high fat diet-fed mice. However, in that study H_2_S was administered for 13 weeks and significantly reduced weight gain and adiposity. It should be noted that in that study H_2_S had no effect on fasting glucose but reduced the increase in glucose after a glucose load. Postprandial glucose level is mainly determined by glucose-induced insulin secretion and the effect of insulin on glucose uptake by the skeletal muscles. The effects of H_2_S on fasting and postprandial glucose could be different. Finally, the study of Cai et al. [21] was performed in high fat diet-fed mice. It is possible that H_2_S has a more marked effect on insulin sensitivity in obese mice in which insulin sensitivity was compromised.

Consistent with our recent study [23], we demonstrated that Na_2_S increased plasma NEFA in vivo as well as glycerol release by adipose tissue ex vivo suggesting a stimulatory effect on lipolysis. As previously demonstrated [23], this effect is mediated by the cAMP-protein kinase A pathway, although the specific mechanism through which Na_2_S increases cAMP in the adipose tissue remains to be established. Insulin inhibits adipose tissue lipolysis by Akt1-mediated phosphorylation and activation of phosphodiesterase 3A which hydrolyzes cAMP and decreases its concentration [24]. Consistently with these data, we observed that insulin decreased plasma NEFA and glycerol in vivo, glycerol release by adipose tissue ex vivo, and cAMP concentration in the adipose tissue both in vivo and ex vivo. We hypothesized that H_2_S could stimulate lipolysis by inhibiting the effect of insulin in the adipose tissue. However, this possibility is unlikely because Na_2_S had no effect on the insulin-induced decrease in plasma NEFA, glycerol, and glucose as well as on the antilipolytic effect of insulin ex vivo. In addition, Na_2_S had no effect on insulin-induced phosphorylation of IRβ, IRS-1, and Akt1. Although Na_2_S itself increased cAMP in the adipose tissue, it had no effect on the insulin-induced decrease in this nucleotide. Together, these results indicate that Na_2_S-induced lipolysis is not mediated by inhibiting insulin sensitivity.

The role of H_2_S in the regulation of insulin signaling and glucose uptake in adipose tissue is controversial. Feng et al. [22] demonstrated that H_2_S (10–1000 μM for 30 min) as well as the CSE substrate L-cysteine reduced basal and insulin-stimulated glucose uptake by cultured rat adipocytes and adipose tissue explants. In contrast, CSE inhibitors increased basal and insulin-stimulated glucose uptake indicating that H_2_S produced under baseline conditions inhibits glucose uptake. Feeding rats with a high fructose diet, which is a commonly used model of insulin resistance, increased CSE expression and H_2_S production in the adipose tissue which correlated with impaired insulin-induced glucose uptake [22]. In addition, tumor necrosis factor-α (TNF α) inhibited glucose uptake in 3T3-L1 adipocytes simultaneously with increasing CSE expression and inhibitors of this enzyme attenuated the effect of TNF-α on insulin-induced glucose uptake [38]. In contrast, NaHS (100 μM) applied for 4 h increased glucose uptake by the same cell line in the absence of insulin by about 20% [21]. Similarly, NaHS or H_2_S solution applied for 24 h increased 2-deoxyglucose uptake in 3T3-L1 adipocytes cultured at low or high glucose concentrations in the presence of insulin and tended to decrease, although not significantly, glucose uptake in the absence of insulin [39]. Consistently, NaHS and H_2_S increased phosphorylation of insulin receptors, phosphoinositide 3-kinase, and Akt. That study also suggested that H_2_S can directly activate insulin receptors, possibly via persulfidation [39]. The stimulatory effect of H_2_S present in the medium for 2 h on glucose uptake and insulin signaling was also observed in 3T3-L1 cells cultured at high glucose concentration which itself impaired insulin sensitivity [40].

The mechanism of these discrepancies is unclear. Although the effect of H_2_S on insulin sensitivity and glucose uptake may differ depending on the animal species and experimental model (adipocyte cell lines, freshly isolated adipocytes, or adipose tissue explants), other possibilities should also be taken into account. First, H_2_S donors tend to reduce glucose uptake if present in the medium for a short time [22] but increase it if applied longer [21,40,41]. Second, H_2_S donors reduce glucose transport to adipocytes and adipose tissue slices obtained from healthy rats [22] but improve it in cells previously exposed to high glucose which is well known to impair insulin signaling [39,40]. In the present study, Na_2_S applied for 15 min reduced basal and insulin-induced glucose uptake by adipose tissue collected from lean rats but had no effect on adipose tissue collected from obese animals in which insulin-induced glucose output was impaired. The mechanism of this effect is unclear at present.

In contrast, Na_2_S_4_ tended to improve insulin signaling, especially in adipose tissue of obese rats. Ex vivo, Na_2_S_4_ increased glucose uptake in the presence of insulin as well as improved insulin-induced phosphorylation of IRS-1 and Akt. Interestingly, in one study [21], the stimulatory effect of NaHS on glucose uptake was abolished by dithiotreitol (DTT). It was suggested that NaHS stimulated glucose uptake by persulfidating peroxisone proliferator-activated receptor-γ and DTT reversed this effect by reducing –SSH to –SH groups. However, the alternative mechanism could also be considered. Inorganic sulfide salts are well known to be oxidized when present for a prolonged period of time in the solution, especially in the presence of cells, to other sulfur species such as polysulfides, thiosulfate, sulfite, and sulfate [41]. H_2_S oxidation in the presence of transition metals is also possible in vivo [42,43]. It could be hypothesized that the stimulatory effect on glucose uptake was mediated by polysulfides originating from NaHS and that DTT abolished this effect by converting them back to H_2_S [21]. This hypothesis could explain why inorganic sulfide salts tend to reduce glucose uptake in the short run but have the opposite effect in longer experiments when spontaneous or cell-mediated oxidation to polysulfides is more likely. In any case, the results of our study suggest different roles of H_2_S and polysulfides in the regulation of insulin signaling in adipose tissue.

Obesity is associated with insulin resistance and impairment of insulin-induced glucose uptake by myocytes and adipocytes. Consistent with these data, we observed that the effect of insulin on glucose uptake, IRS-1, and Akt phosphorylation was impaired in obese rats. At the molecular level, insulin resistance is often associated with impaired tyrosine phosphorylation of IRS-1 despite normal activation of the insulin receptor. Reduced insulin-induced tyrosine phosphorylation of IRS-1 may result from its enhanced phosphorylation at serine and/or threonine residues by various serine-threonine protein kinases such as protein kinases C, inhibitor κB kinase, mitogen-activated protein kinases, Rho kinase, etc. [44]. Currently, the effect of insulin on IRβ phosphorylation was intact in obese rats which would be consistent with this mechanism. Nevertheless, the mechanism of impaired tyrosine phosphorylation of IRS-1 remains to be established. The mechanism through which polysulfides improve IRS-1 phosphorylation will also be the subject of future research. The interaction between persufidation and phosphorylation has been demonstrated for other proteins such as endothelial NO synthase [45]. Alternatively, polysulfides could improve tyrosine phosphorylation of IRS-1 by targeting protein kinases phosphorylating its serine/threonine residues.

Despite impaired insulin signaling and insulin-induced glucose uptake, the effect of insulin on lipolysis was intact in obese rats. It is well known that the antilipolytic effect of insulin is less sensitive to insulin deficiency or insulin resistance than its effect on glucose metabolism. Consequently, enhanced lipolysis and ketogenesis are observed in type 1 diabetes associated with severe or complete insulin deficiency but are unlikely in type 2 diabetes even in patients with severe hyperglycemia. It should be noticed that despite impaired IRS-1 and Akt phosphorylation, the effect of insulin on cAMP, which is involved in the suppression of lipolysis but not in the stimulation of glucose uptake, was intact in obese rats. In addition, fasting glucose level was normal in obese rats. Again, a lower level of insulin signaling is required for suppressing hepatic glucose output—the main determinant of fasting glucose levels—than for stimulating glucose uptake. Interestingly, we observed that this model of obesity was associated with impaired glucose tolerance despite fasting normoglycemia; the constellation characteristic for prediabetes (unpublished observation).

Obesity is characterized by stimulation of baseline adipose tissue lipolysis which was confirmed by higher fasting NEFA and glycerol levels. The results of this study suggest that this was not the consequence of insulin resistance of the adipose tissue. The possible mechanisms of enhanced lipolysis despite the preserved antilipolytic effect of insulin may include increased expression of triglyceride-hydrolyzing enzymes, increased adrenergic activity, reduced ghrelin, and adiponectin concentrations reduced expression/activity of AMPK-activated protein kinase, and abnormal cortisol metabolism [24,46].

There are several limitations of the present study. First, we examined the effect of Na_2_S and Na_2_S_4_ only in the short run. Chronic effects of H_2_S and H_2_S_n_ may be different than acute ones. However, H_2_S and H_2_S_n_ may be interconverted in vivo in redox reactions. Acute administration of the respective donors minimizes the risk of interconversion and allows to address the effect of different RSS more specifically. Second, inorganic sulfide salts provide H_2_S in high quantities and for a short time. Synthetic slow-releasing donors such as GYY4137 are considered more physiological. However, Na_2_S and Na_2_S_n_ used by us are chemically related H_2_S and H_2_S_n_ donors, respectively, making their effects more comparable whereas there are no chemically related H_2_S_n_-releasing counterparts for currently used slow-releasing H_2_S donors. Third, in ex vivo experiments we used only one depot of visceral adipose tissue and it is well recognized that different parts of visceral and subcutaneous adipose tissue have different metabolic characteristics. Fourth, we examined the effect of RSS on insulin sensitivity only in adipose tissue and the effects on other insulin target tissues could be different. Nevertheless, we believe that the results presented here provide new data about the effect of RSS on adipose tissue metabolism.

## 5. Conclusions

In conclusion, acutely administered H_2_S and polysulfide donors have no effect on plasma glucose and insulin concentrations in the fasting state, suggesting little or no effects on insulin secretion and sensitivity. H_2_S and polysulfides have no effect on the glucose-lowering effect of insulin in vivo and on the antilipolytic effect of this hormone either in vivo or ex vivo. However, polysulfides, but not H_2_S, improve insulin signaling and insulin-induced glucose uptake in adipose tissue of obese rats suggesting that various reactive sulfur species have different effects on insulin sensitivity and that increasing polysulfide concentration or signaling may be a promising therapeutic approach in diseases associated with insulin resistance.

## Figures and Tables

**Table 1:** Effect of obesity on metabolic parameters, H_2_S and polysulfide production in adipose tissue.

Metabolic Parameter	Control	Obese
Body weight (g)	228 ± 18	278 ± 20 **
Weight gain (g)	18 ± 4	82 ± 7
Triglycerides (mM)	0.86 ± 0.08	0.88 ± 0.09
Total cholesterol (mM)	2.18 ± 0.27	2.25 ± 0.32
NEFA (μM)	361 ± 28	744 ± 49 ***
Glycerol (μM)	177 ± 18	261 ± 22 ***
Insulin (μU/mL)	22.2 ± 2.3	23.2 ± 2.7
Glucose (mM)	4.22 ± 0.51	4.44 ± 0.58

** *p* < 0.01, *** *p* < 0.001 vs. control group.

## Data Availability

Original data are available from the corresponding author on request.
